# A new classification of talocalcaneal coalitions based on computed tomography for operative planning

**DOI:** 10.1186/s12891-021-04567-0

**Published:** 2021-08-11

**Authors:** Anhong Wang, Weili Shi, Lixiang Gao, Linxin Chen, Xing Xie, Feng Zhao, Yanbin Pi, Chen Jiao, Yuelin Hu, Dong Jiang, Qinwei Guo

**Affiliations:** 1 Department of Sports Medicine, Peking University Third Hospital. Institute of Sports Medicine of Peking University. Beijing Key Laboratory of Sports Injuries, 49 North Garden Road, Haidian District, Beijing, 100191 China; 2 Department of Radiology, Peking University Third Hospital, Beijing, China

**Keywords:** Talocalcaneal coalition, Classification, Articular facet, Computed tomography

## Abstract

**Background:**

Current classifications emphasize the morphology of the coalition, however, subtalar joint facets involved should also be emphasized.

**Objective:**

The objective of this study was to develop a new classification system based on the articular facets involved to cover all coalitions and guide operative planning.

**Methods:**

Patients were diagnosed with talocalcaneal coalition using a CT scan, between January 2009 and February 2021. The coalition was classified into four main types according to the shape and nature of the coalition: I, inferiorly overgrown talus or superiorly overgrown calcaneus; II, both talus and calcaneus overgrew; III, coalition with an accessory ossicle; IV, complete osseous coalition (I-III types are non-osseous coalition). Then each type was further divided into three subtypes according to the articular facets involved. A, the coalition involving the anterior facets; M, the coalition involving the middle facets, and P, the coalition involving the posterior facets. Interobserver reliability was measured at the main type (based on nature and shape) and subtype (articular facet involved) using weighted Kappa.

*Results* There were 106 patients (108 ft) included in this study. Overall, 8 ft (7.5%) were classified as type I, 75 ft (69.4%) as type II, 7 ft (6.5%) as type III, and 18 ft (16.7%) as type IV. Twenty-nine coalitions (26.9%) involved the posterior facets only (subtype-P), 74 coalitions (68.5%) involved both the middle and posterior facets (subtype-MP), and five coalitions (4.6%) simultaneously involved the anterior, middle, and posterior facets (subtype-AMP). Type II-MP coalition was the most common. The value of weighted Kappa for the main type was 0.93 (95%CI 0.86–0.99) (*p*<0.001), and the value for the subtype was 0.78 (95%CI 0.66–0.91) (*p*<0.001).

**Conclusion:**

A new classification system of the talocalcaneal coalition to facilitate operative planning was developed.

## Introduction

Talocalcaneal coalition is the abnormal bridge between talus and calcaneus. It is a type of tarsal coalitions and is attributed to the failure of differentiation and segmentation in the primitive mesenchyme [1–3]. Talocalcaneal coalition is a significant cause of hindfoot pain, limited motion, and a valgus heel [4–6]. The talocalcaneal coalition can be divided into syndesmosis, synchondrosis, and synostosis by its nature.

A plain radiograph is still the first choice to evaluate talocalcaneal coalition. A weight-bearing anterior-posterior, lateral radiograph, and Harris-heel view are commonly used [7, 8]. A feature of the C-sign, which formed by the outline of the talar dome and the inferior outline of the sustentaculum tali in the lateral radiograph may be indicated. However, the diagnosis of the talocalcaneal coalition is difficult depending on the plain radiograph and the C-sign lacks sensitivity [9].

The normal subtalar joint is divided into the anterior, middle, and posterior joint. The talocalcaneal coalition is thought to be most commonly developed in the middle part of the subtalar joint [10–12]. The middle and anterior facets were both concave on the calcaneus and fused anterior and middle articular facets were seen more frequently [13, 14]. The bridged anterior and middle joint facets were considered an integration because they were continuous with the talocalcaneonavicular joint [14, 15]. The tarsal sinus, lying lateral to open space the middle joint facet, separates the posterior and middle facet joints. It is the boundary to distinguish the middle from the posterior subtalar joint facets. For the complicated anatomy, plain radiographs cannot determine the type and location of the coalition, while computed tomography (CT) enables a good depiction of the subtalar anatomy, to effectively determine the size and shape, and also allows distinguishing between osseous and non-osseous coalitions [2, 10, 14–16]. CT has been regarded as the standard diagnostic modality and is helpful to preoperative planning. Rozansky et al. [17] in 2010 classified the talocalcaneal coalition into five types based on the coalition nature, location, and facet joint orientation. After that, Sanghyeok Lim et al. [18] in 2013 used CT and MRI to evaluate the coalition and developed a classification according to the coalition nature and shape. However, besides coalition nature and shape, subtalar joint facets involved should also be emphasized.

The purpose of this study is to develop a new classification system for operative planning based on the morphology, nature of the coalition, and the subtalar articular facets involved.

## Methods and materials

The retrospective study was approved by the Board of Research Ethics. Patients were identified through the clinical medical record system in our hospital using the keywords of “talocalcaneal coalition”, “tarsal coalition” or “coalition”, between January 2009 and February 2021. One hundred and twenty-one subjects were identified. After that, patients with talocalcaneal coalition confirmed using CT scan were included. Exclusion criteria included talonavicular or calcaneonavicular coalition and patients without a CT scan.

CT examinations were performed using a 64-slices helical CT (GE, USA). All the CT images were reviewed by the corresponding author (with 18 years of orthopedic sports medicine experience). We distinguished the middle from the posterior subtalar joint facet by the boundary of the tarsal sinus in the coronary planes of CT, in the Picture Archiving Communication System (PACS). Diagnosis criteria were as follows: osseous coalitions were confirmed by the presence of a bony bridge; nonosseous coalitions were confirmed by manifestations of narrowing of the facet with marginal cortical irregularity as Kumar et al. depicted [19] and overgrown talus or calcaneus was discovered. Besides, a 3D construction image was obtained to show the details of the abnormal bar [17].

**Fig. 1:**
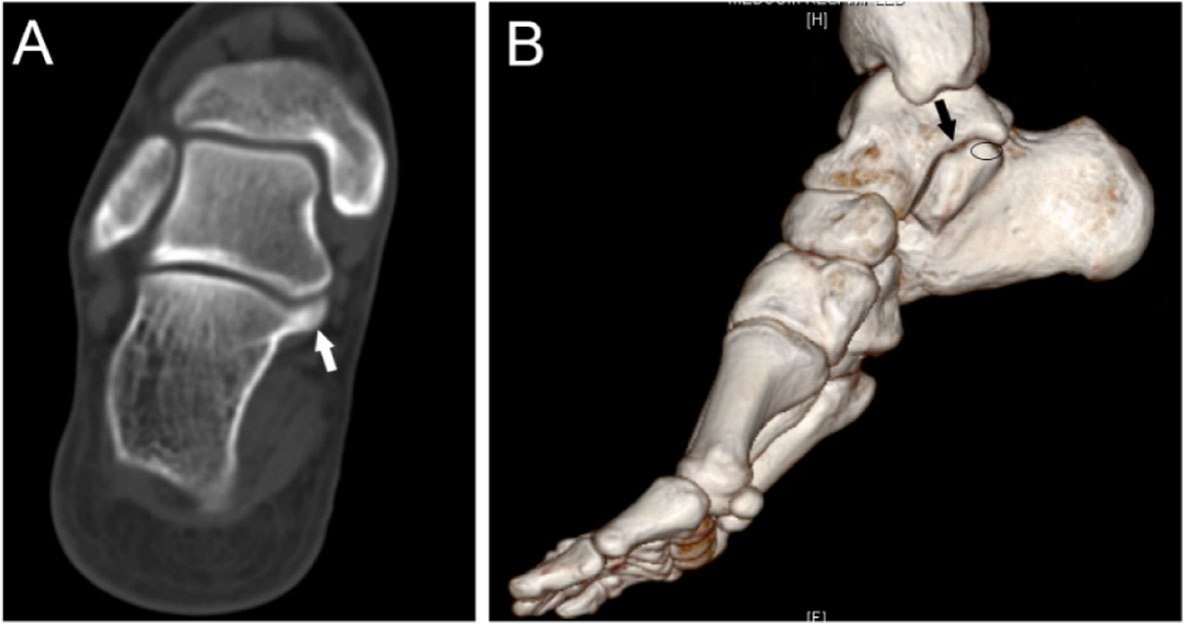
25-year-old man with an I-P type coalition involving the posterior facet only. (A) Superiorly overgrown calcaneus (white arrow) covers the articular facet of the talus. (B) 3D construction image shows the open of sinus tarsal (black arrow) between the sustentaculum tali and the coalition (oval)

**Fig. 2:**
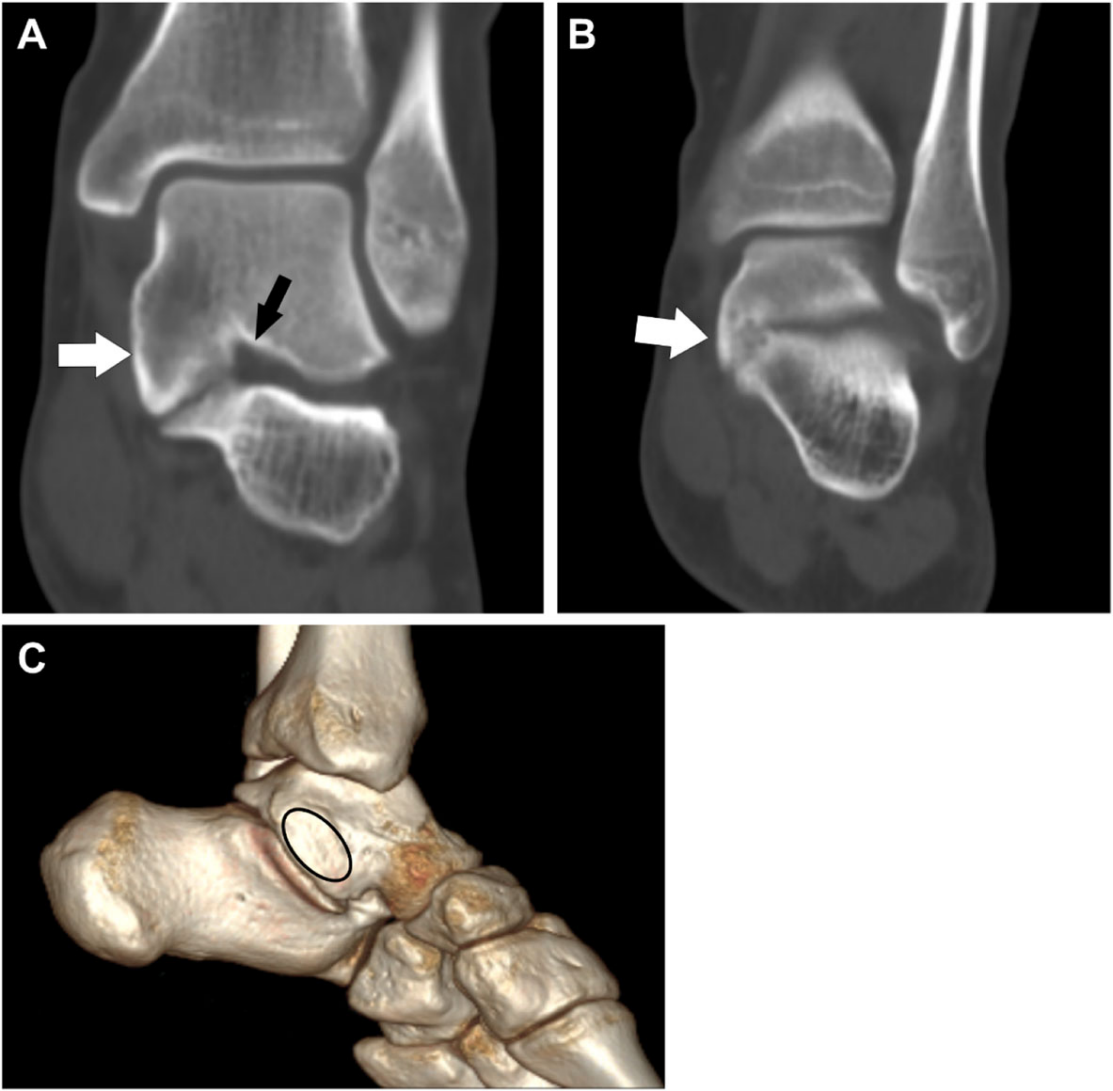
22-year-old male with an I-MP type coalition involving both middle and posterior facets. (A) The tarsal sinus (black arrow) can be seen. A significantly overgrown talus (white arrow) covers the calcaneus. The marginal cortical irregularity of the calcaneus is also noted. (B) The tarsal sinus cannot be seen and only the posterior facet is shown (white arrow). The two images (A, B) indicate that the coalition involved both the middle and posterior facets. A significantly overgrown talus (white arrow) covers the calcaneus. The marginal cortical irregularity of the calcaneus is also noted. (C) 3D reconstruction image shows that the overgrown talus covers the calcaneus like a shingle (oval) without the open of sinus tarsal

**Fig. 3:**
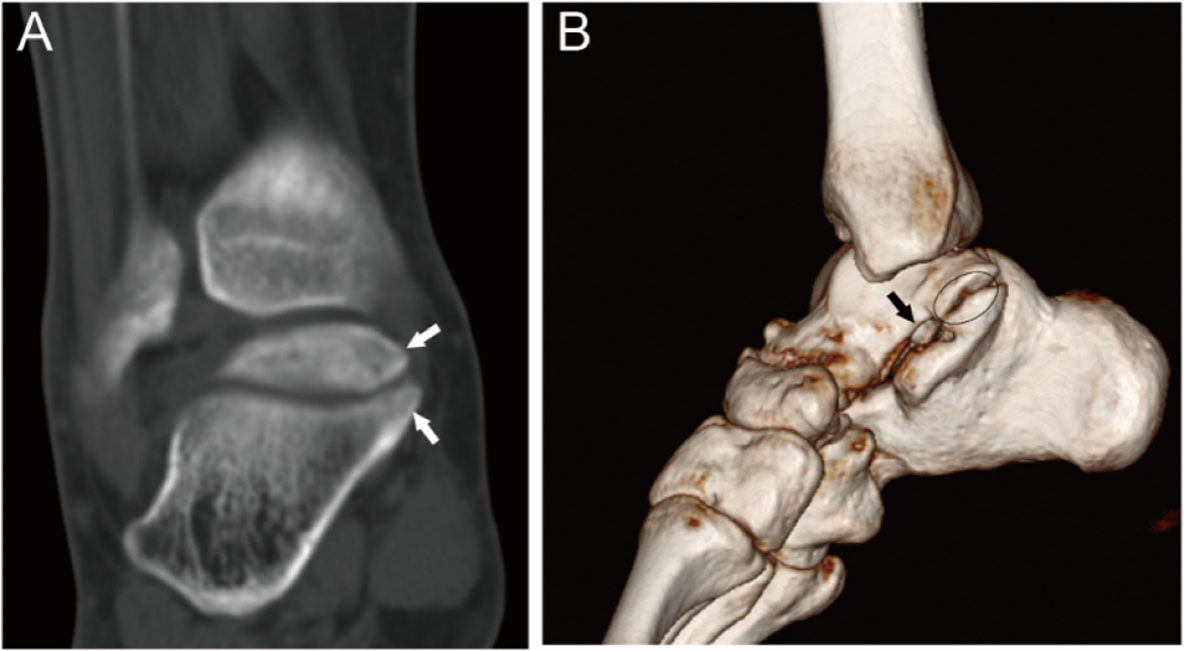
21-year-old man with an II-P type coalition involving the posterior facet only. (A) The tarsal sinus cannot be seen; both talus and calcaneus (white arrows) overgrow to adapt to each other. (B) 3D construction shows the open of sinus tarsal (black arrow); A significant coalition (oval) can be identified

**Fig. 4:**
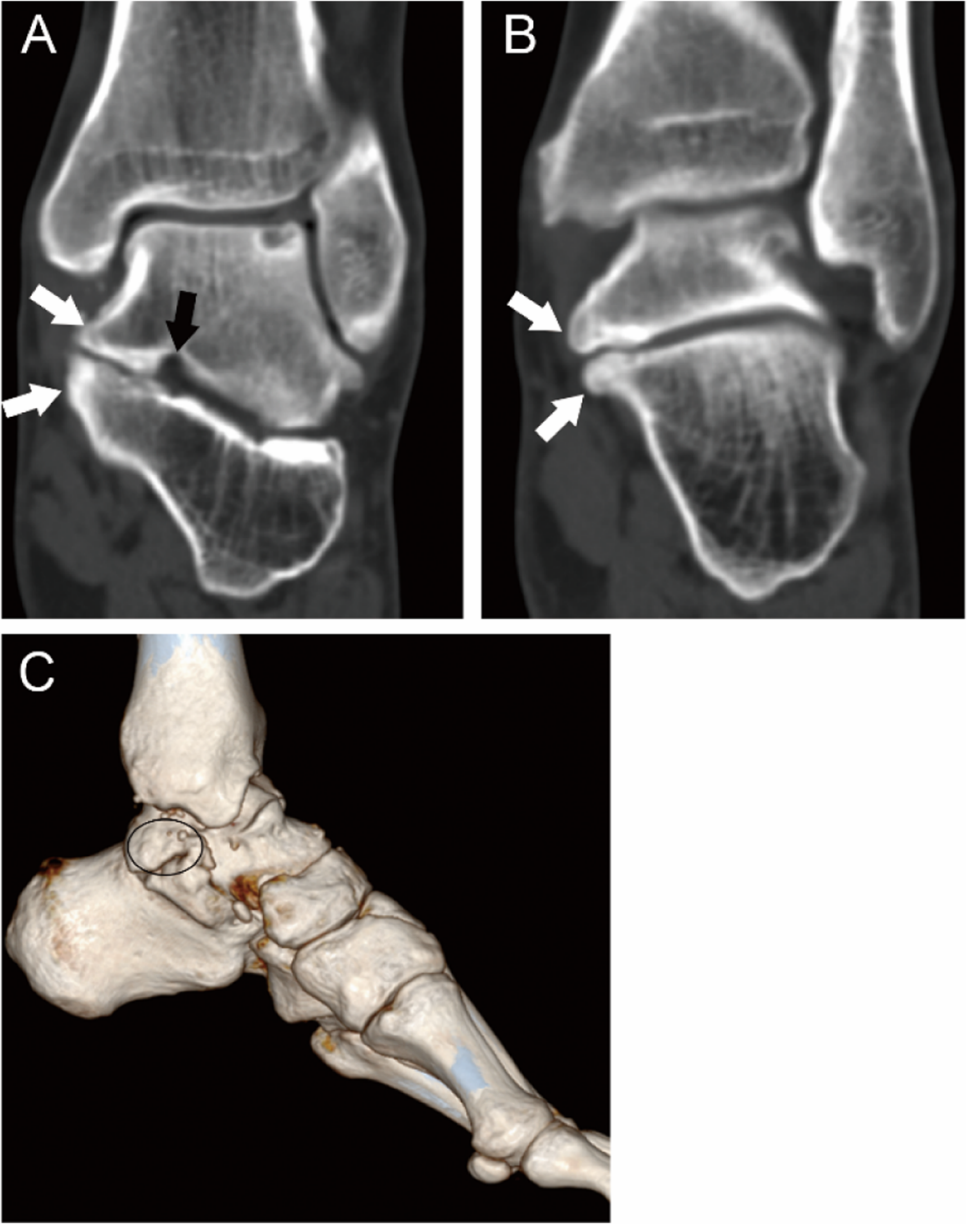
34-year-old man with an II-MP type coalition involving both the middle and posterior facets. (A, B) Both the talus and calcaneus overgrow (white arrows) and cortical irregularity of the calcaneus and talus is obvious. The tarsal sinus (black arrow) can be seen, which indicates that the coalition involves the middle facet (fig.-A), however, the tarsal sinus is not shown behind that plane (fig.-B), indicating that the coalition affects the posterior facet. (C) 3D construction image shows that both the talus and calcaneus (oval) overgrow and cover each other. The open of sinus tarsal is not found

**Fig. 5:**
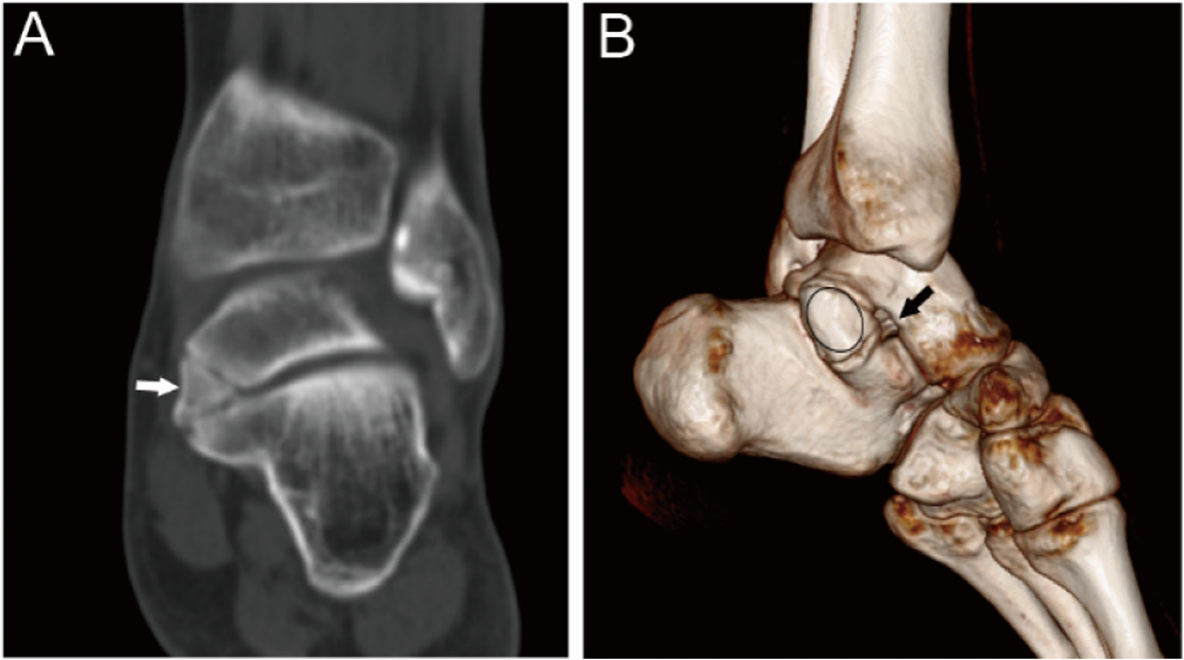
24-year-old male with an III-P type coalition involving the posterior facet only. (A) An independent coalition with an accessory ossicle (white arrow) locates at the medial and inferior aspect of the talus and superior aspect of the calcaneus. The sclerosis was not found at the border between the ossicle and calcaneus or talus. (B) 3D reconstruction image shows the open of sinus tarsal (black arrow), and an accessory ossicle (oval)

**Fig. 6:**
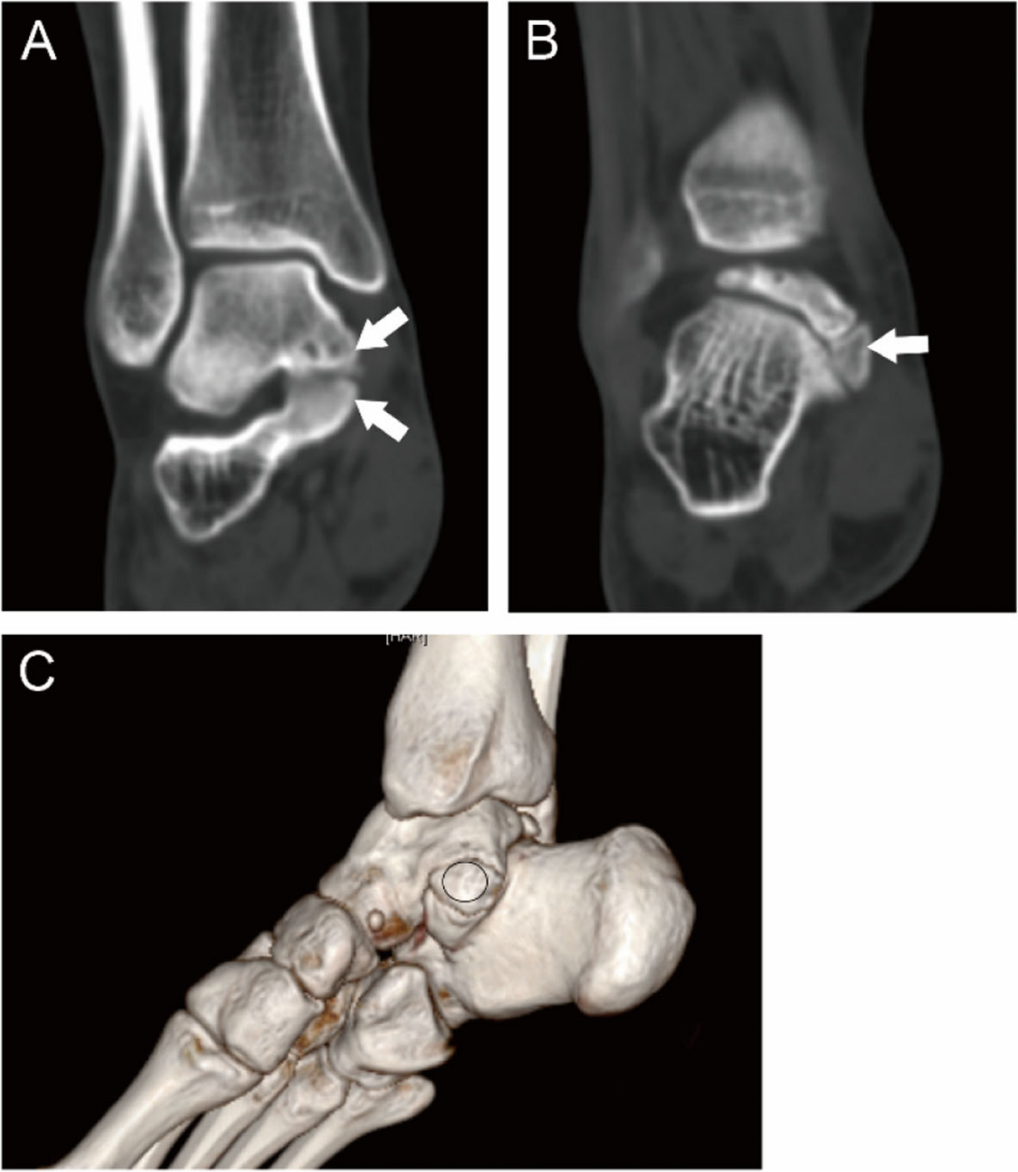
35-year-old female with an III-MP type coalition involving both the middle and posterior facets. (A) The tarsal sinus (black arrow) can be seen and both overgrown talus and calcaneus (white arrows). (B) An accessory ossicle (white arrow) locates at the medial and inferior aspect of the talus and superior aspect of the calcaneus. The sclerosis is not found at the border between the ossicle and calcaneus or talus. (C) 3D reconstruction image shows that an accessory ossicle (circle) separates the talus and calcaneus

**Fig. 7:**
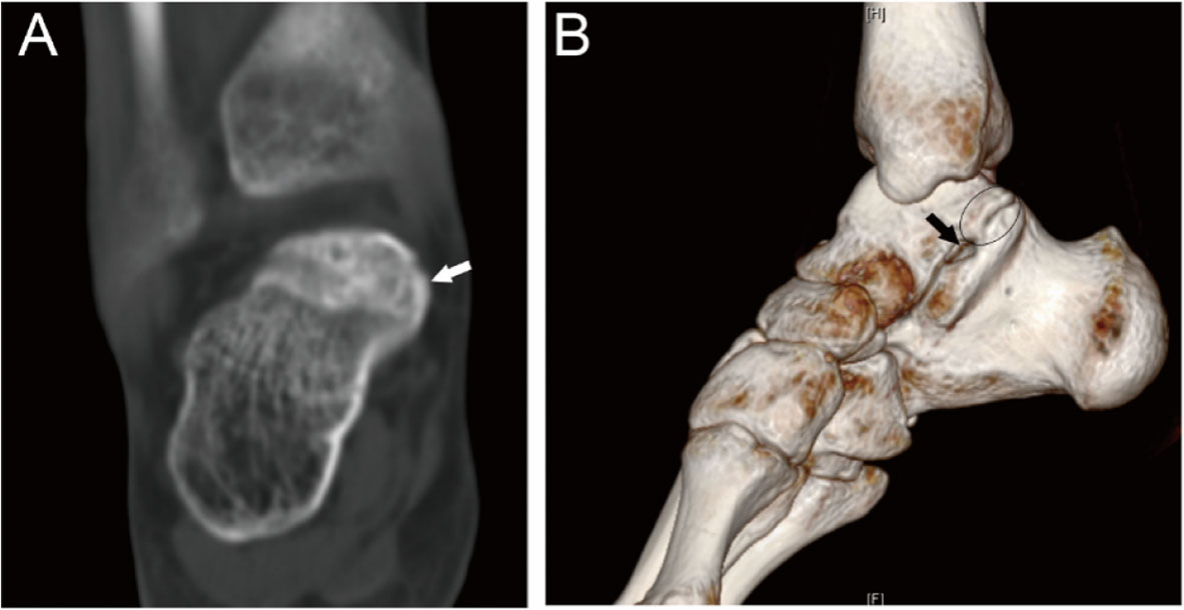
29-year-old female with an IV-P type coalition involving the posterior facet only. (A) Fused talus and calcaneus form a complete osseous coalition (white arrow) involving the posterior facet and cannot be torn apart. (B) 3D construction image shows a complete osseous (oval) bridge between talus and calcaneus. The open of sinus tarsal (black arrow) can also be seen

**Fig. 8:**
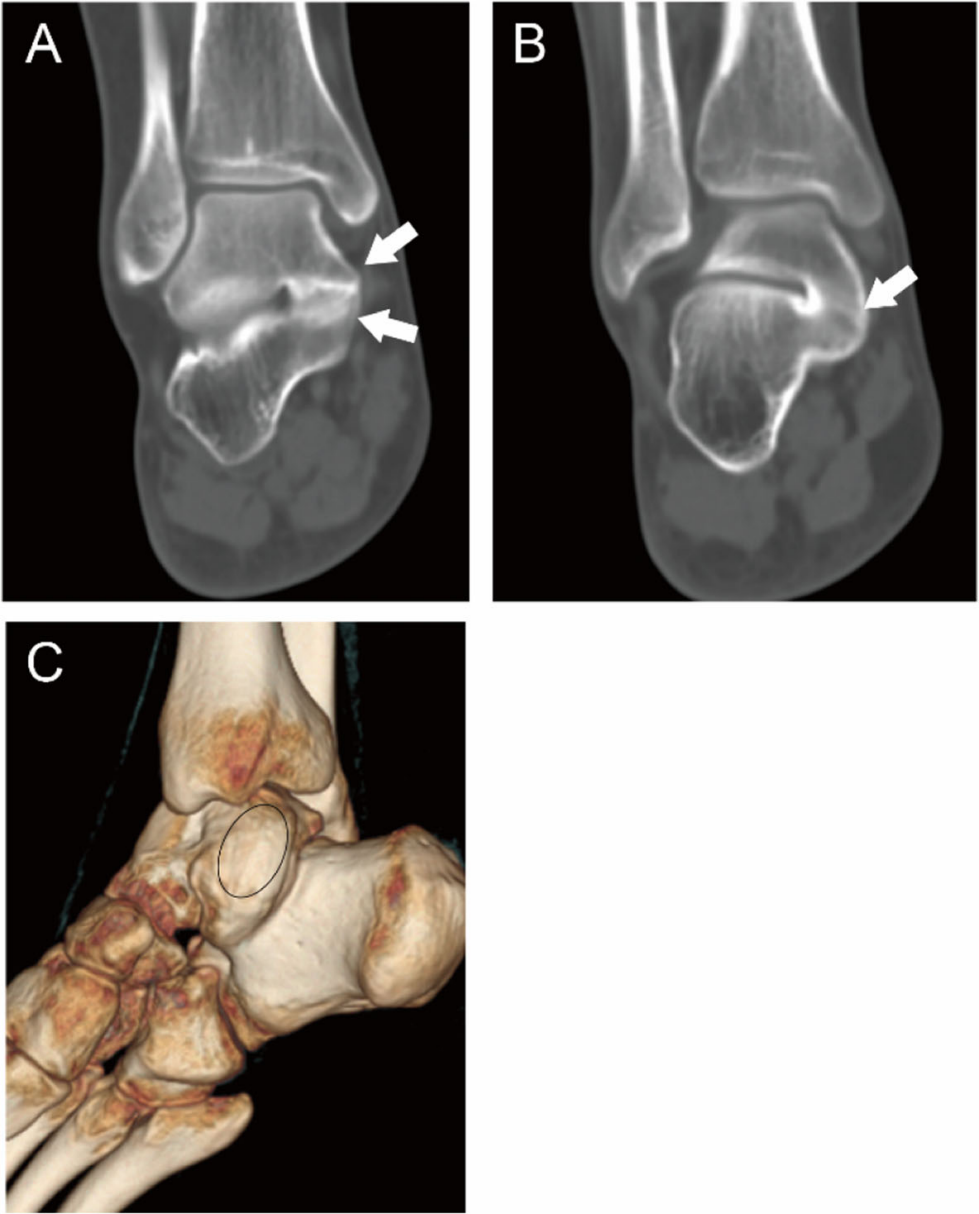
23-year-old man with an IV-MP type coalition involving both the middle and posterior facets. (A, B) The tarsal sinus (black arrow) can be seen and both the talus and calcaneus (white arrows) overgrow and cover each other (A). A complete osseous coalition (white arrow) can be seen in the posterior facet (B). (C) 3D construction image shows that the talus and calcaneus fuse together (oval) without the open of tarsal sinus

**Fig. 9:**
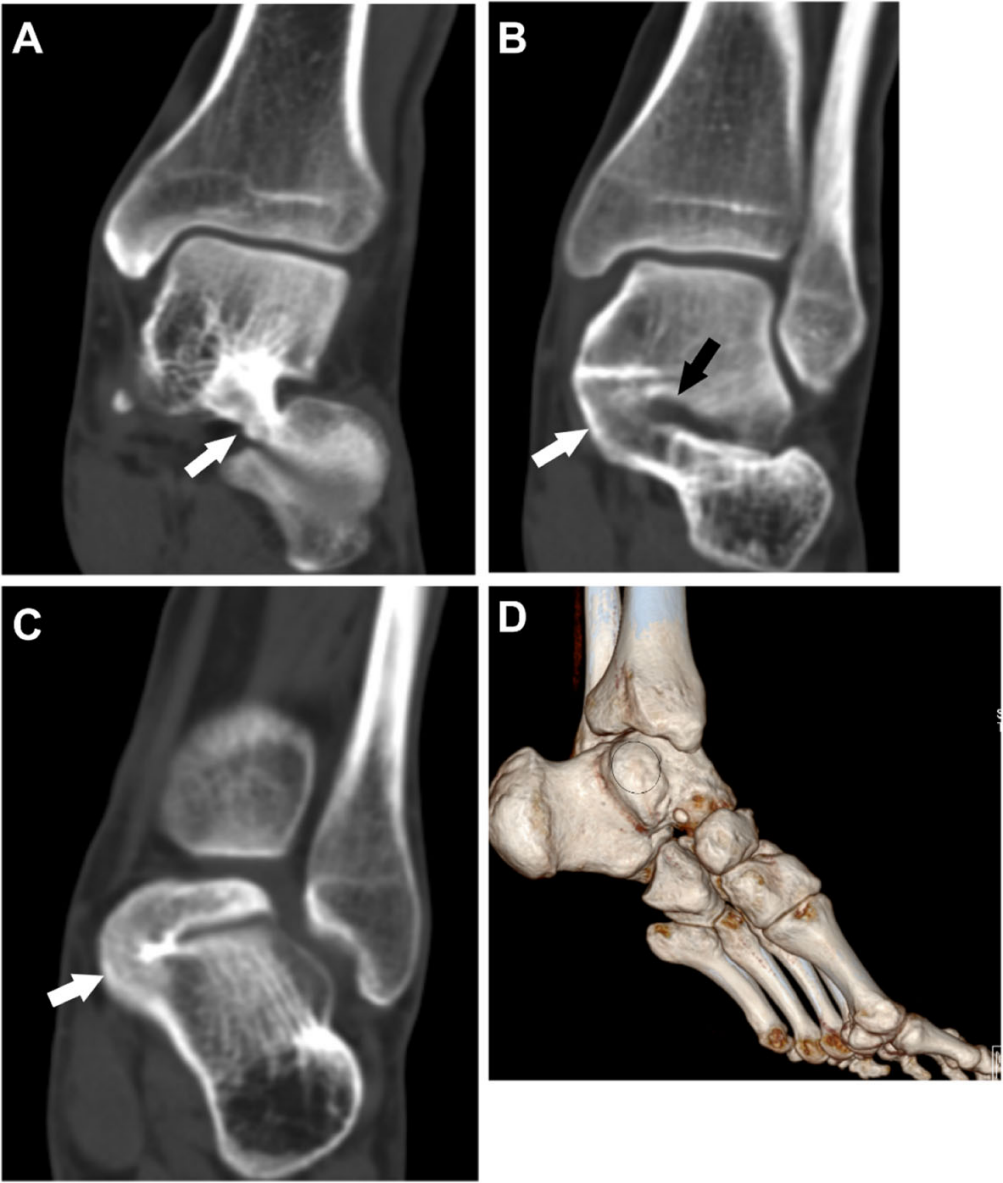
57-year-old man with an IV-AMP type coalition involving the anterior, middle and posterior facets. (A, B, C) Fused talus and calcaneus form a complete osseous coalition (white arrow) involving the anterior, middle and posterior facets. (B) The tarsal sinus (black arrow) can be seen. (C) 3D construction image shows that the talus and calcaneus fuse together (oval) without the open of tarsal sinus

The coalitions were classified into four main types according to the shape and nature of the coalition: I, inferiorly overgrown talus or superiorly overgrown calcaneus; II, both talus and calcaneus overgrew; III, a coalition with an accessory ossicle; (I-III types are non-osseous coalition) IV, complete osseous coalition. On the coronal images of CT, the shape and nature of the coalitions could be observed. Then each type was further divided into three subtypes according to the articular facets involved. A, the coalition involving the anterior facets; On the coronal images of CT, the coalition could only be found anterior to the images of the tarsal sinus for this subtype. M, the coalition involving the middle facets. The subtypes could be identified through the coronal images of CT of the tarsal sinus. P, the coalition involving the posterior facets. On the coronal images of CT, the coalition could only be found posterior to the images of the tarsal sinus. For the coalitions involving the posterior facets only, the open of the sinus tarsal was obvious on a 3D reconstruction image. While for the coalitions involving the middle and posterior facets, the open of the sinus tarsal could not be seen on a 3D reconstruction image (Figs. 1, 2, 3, 4, 5, 6, 7, 8 and 9).

The images were first evaluated by Dr. Guo with over twenty years of clinical and radiological experience in foot and ankle to base the classification. To evaluate the interobserver agreement, a radiologist (Dr. Gao) with over 5 years of radiological experience in musculoskeletal images, then independently reviewed the images of 108 cases after 2 weeks of training session. The 2 observers were blinded to the classification made by each other.

Data were calculated using statistical software (Excel; Microsoft Corporation, USA). The quantitative data were presented as mean ± standard deviation unless otherwise specified. The interobserver agreement on this classification was analyzed using weighted Kappa and the confidence interval (CI) was also calculated. Agreement was measured at the main type (based on nature and shape) and subtype (articular facet involved). A value of Kappa that ranged from 0.81 to 1.00 was defined as almost perfect reliability, 0.61 to 0.80 as substantial, 0.41 to 0.60 as moderate, 0.21 to 0.40 as fair, and 0.00 to 0.20 as slight [20]. Statistical analysis was performed using the Statistical Package for the Social Science (SPSS 25.0) software. Statistical significance was established at *p* < 0.05.

## Results

A total of 106 patients (108 ft) with talocalcaneal coalition were included in this study, of which 2 patients had bilateral coalitions. There were 76 male patients (70.4%) and 30 females (28.8%). Sixty-four right feet (59.3%) were affected while 44 coalitions (40.7%) were on the left feet. The average patient age was 29.8 ± 11.0 years (range 12–60 years).

Overall, 8 ft (7.5%) were classified as type I, 75 ft (69.4%) as type II, 7 ft (6.5%) as type III, and 18 ft (16.7%) as type IV. All coalitions involved the posterior facets. Twenty-nine coalitions (26.9%) involved the posterior facets only, 74 coalitions (68.5%) involved both the middle and posterior facets, and five coalitions (4.6%) simultaneously involved the anterior, middle, and posterior facets (Table 1). We didn’t find any coalition involving the anterior facets in type I, II, and III. We analyzed the interobserver agreement for the main type (shape and nature) and subtype (Articular facet involved). The value of weighted Kappa for the main type was 0.93 (95%CI 0.86–0.99) (*p*<0.001), and the value for the subtype was 0.78 (95%CI 0.66–0.91) (*p*<0.001) (Table 2).

**Table 1:** The classification of the talocalcaneal coalition

Classification^a^	Num. of feet	Percentage
I		
I-P	2	1.9%
I-MP	6	5.6%
II		
II-P	20	18.5%
II-MP	55	50.9%
III		
III-P	4	3.7%
III-MP	3	2.8%
IV		
IV-P	3	2.8%
IV-MP	10	9.3%
IV-AMP	5	4.6%
Total	108	100%

^a^Type I, inferiorly overgrown talus or superiorly overgrown calcaneus; II, both talus and calcaneus overgrew; III, coalition with an accessory ossicle; (I-III types are non-osseous coalition) IV, complete osseous coalition. Then each type was further divided into three subtypes according to the articular facets involved. A, the coalition involving the anterior facets; M, the coalition involving the middle facets, and P, the coalition involving the posterior facets

**Table 2:** The interobserver agreement on this classification of talocalcaneal coalition

	Kappa	95% CI	*p*-value
Shape and nature	0.93	0.86–0.99	<0.001
Articular facet involved	0.78	0.66–0.91	<0.001

*CI* confidence interval.

## Discussion

Based on the shape, nature of the coalitions, and the articular facets that coalitions involved, we devised a new classification system through analyzing the large sample of cases and found the coalition with both talus and calcaneus overgrew, involving the posterior and middle subtalar joint facets, was most common.

Rozansky et al. [17] classified the talocalcaneal coalition into five types with its nature and shape, which can provide details for surgical resection. The posterior coalitions were defined as Type V in their study. However, the involved articular facets were not discussed among their type I to type IV coalitions, and the coalition with an accessory ossicle was not reported. Sanghyeok Lim et al. [18] analyzed the characteristics of talocalcaneal coalition among 70 ft, and reported the coalition with an accessory ossicle called as a fracture fragment. The shape and direction of coalition were emphasized in their classification. However, the location of the coalition was not included. In this study, we firstly classified the coalition according to its nature and shape into four main types, and then each type was divided into three subtypes according to its articular facets involved. By this classification, all kinds of coalitions could be covered, including the isolated anterior or middle facet coalition that we didn’t report. This classification provides information about the location, nature, and orientation of talocalcaneal coalition, which is important for surgical excision. Almost perfect and substantial interobserver reliability were achieved for the main type and subtype, respectively, which indicated it was reliable for assessing the talocalcaneal coalition.

Type II-MP was the most common type, comprising 50.9% of the coalitions, manifesting with both overgrown talus and calcaneus. We found only 8 coalitions (7.5%) of type I (two I-P types and 6 I-MP types). It was lower than 23 coalitions (33%) in Sanghyeok Lim et al’s study [18]. Type I and II showed the coalition orientation (sloping up or down, or horizontal) which was helpful to find the fibrocartilage or fibrous line between the talar part and calcaneal part to guide to operative resection, particularly during arthroscopic resection. Seong Jong Yun and his colleagues [21] reported that 15 of 54 (27.8%) feet showed talocalcaneal coalitions with an accessory ossicle. They regarded the accessory bone as os sustentaculum, forming when the accessory ossification center ossified, at the medial and posterosuperior aspects of the sustentaculum tali and they believed that the accessory bone may be a cause of bone marrow edema and pain in osteoarthritis. Sanghyeok Lim et al. [18] regarded this accessory ossicle as a fracture fragment and they found a coalition with a “fracture fragment” in 17 of 70 ft (24%). We found 7 coalitions (6.5%) with an accessory ossicle (Type III) in this study. We also thought the coalition might be an accessory ossicle but not a fracture fragment, because the sclerosis of nonunion was not found in the ossicle by CT.

A complete osseous coalition may be difficult for resection for it’s hard to identify the borderline of the coalition, particularly in arthroscopic surgery. There were 18 ft (16.7%) identified as complete osseous coalitions (Type IV) in this study. This was in line with the study of Rozansky et al. [17]. However, Wael Aldahshan et al. [22] reported 8 complete bony coalitions (40%) while Amir Khoshbin et al. [23] also found 5 complete osseous coalitions(38.3%). But in Sanghyeok Lim et al.’s study [18], there were only 2 complete synostosis coalitions (3%)^18^. The difference may lie in the different sample sizes.

The subtalar middle facet was most commonly involved while the posterior facet coalition was rare as reported in some studies [14, 16, 24]. Soon Hyuck Lee et al. [25] reported recently that the prevalence of the talocalcaneal coalition in the middle and posterior subtalar facets was 27%, while 68% of coalitions involved the posterior facet only. Seong Jong Yun et al. [21] reported that the prevalence of subtalar posterior facet coalition (34.6%) was higher than the middle facet coalition (9.9%) in 81 patients. Scranton, P. E. et al. [26] reported 10 posterior coalitions (55.6%) in 18 ft. These studies indicated that coalition in the posterior facet was not as rare as long believed and took up a great part of the talocalcaneal coalition. In the current study, we found that all coalitions (100%) involved the posterior articular facet, while 73.2% of coalitions involved both the middle and posterior articular facets. The finding that the coalition involving the posterior facets was more than the coalition involving the middle facets, was consistent with the studies of Soon Hyuck Lee et al. [25] and Seong Jong Yun et al. [21]. However, the coalition only involved the middle facet was not found in our case series. The studies about the coalition involving the anterior facets were rare [16, 27], and we found only five coalitions (4.6%) that involved the anterior facets.

Rozansky et al. [17] depicted the features of coalitions on the 3D construction image, however, they didn’t emphasize the open of the tarsal sinus. In the current study, the open of the tarsal sinus could be found in the subtype-P coalitions on a 3D construction image, while for subtype-MP and AMP, it could not be found. So, we can also distinguish the facets that the coalitions involve from a 3D construction image.

The first-line strategy for symptomatic talocalcaneal coalitions is conservative treatment [8, 11]. Coalition resection is recommended if non-operative treatment failed. Traditional open techniques may prolong hospitalization for wound management and pain control [28]. An open technique [29, 30] is often performed with an incision over the sustentaculum tali, and then, identifying the bridge edge through the talonavicular joint anteriorly and the residual talocalcaneal joint. Finally, the coalition is resected until the articular cartilage is visible. Arthroscopy has gained popularity recently and several authors reported good results after endoscopic coalition resection [22, 31, 32].

For the subtype-P coalitions, the excision is enough until healthy cartilage of the posterior subtalar joint is visualized. While for subtype-MP coalitions, the excision should be extended anteriorly to the medial open of the tarsal sinus in an open technique. It is similar to arthroscopic surgery, in which the flexor hallucis longus (FHL) is an important landmark [33, 34]. The excision under the arthroscope should be extended medially, according to the non-osseous coalitions of types I-III. In type IV (osseous coalition), an important landmark that can help identify the location of the subtalar joint is the posterior talofibular ligament [33].

## Conclusion

A new classification system of the talocalcaneal coalition to facilitate operative planning was developed.

### Limitations

The limitation of this study lies in its retrospective nature. However, as we know, this is the largest sample size currently reported and may make up for this shortcoming. Some studies referred to the coalition that only involved the middle facet. However, the coalition only involving the anterior facet or middle facet was not found in this case series.

## Data Availability

The dataset analysed during the current study is available from the corresponding author on reasonable request.
